# The directed evolution of ligand specificity in a GPCR and the unequal contributions of efficacy and affinity

**DOI:** 10.1038/s41598-017-16332-2

**Published:** 2017-11-22

**Authors:** Raphaël B. Di Roberto, Belinda Chang, Sergio G. Peisajovich

**Affiliations:** Department of Cell and Systems Biology, University of Toronto 25 Harbord Street, Toronto, ON M5S 3G5 Canada

## Abstract

G protein-coupled receptors (GPCRs) must discriminate between hundreds of related signal molecules. In order to better understand how GPCR specificity can arise from a common promiscuous ancestor, we used laboratory evolution to invert the specificity of the *Saccharomyces cerevisiae* mating receptor Ste2. This GPCR normally responds weakly to the pheromone of the related species *Kluyveromyces lactis*, though we previously showed that mutation N216S is sufficient to make this receptor promiscuous. Here, we found that three additional substitutions, A265T, Y266F and P290Q, can act together to confer a novel specificity for *K*. *lactis* pheromone. Unlike wild-type Ste2, this new variant does not rely on differences in binding affinity to discriminate against its non-preferred ligand. Instead, the mutation P290Q is critical for suppressing the efficacy of the native pheromone. These two alternative methods of ligand discrimination were mapped to specific amino acid positions on the peptide pheromones. Our work demonstrates that changes in ligand efficacy can drive changes in GPCR specificity, thus obviating the need for extensive binding pocket re-modeling.

## Introduction

Communications within and between cells are an essential feature of life. As organisms evolved, the complexity of cell signaling networks has increased dramatically^1^. Protein receptors today must distinguish between countless signaling molecules, many of them displaying similar structures. This is especially true for G protein-coupled receptors (GPCRs), the large family of seven-transmembrane receptors involved in neurotransmission, chemokine recognition, vision and olfactory sensing. Changes in GPCR specificity can have dramatic consequences in phenotype, causing disease^2,3^ or major evolutionary shifts^4^. Due to their pharmacological importance, much research has been devoted to understanding how GPCRs interact with their cognate ligands and how their binding specificity can be altered. High-resolution crystal structures of GPCR-ligand complexes have been invaluable for revealing the precise electrostatic interactions that are involved in binding^5,6^. However, much less is known about how specificity can be altered. Phylogenetic analyses have been used to identify specificity-determining positions (SDPs) which can then be validated in the laboratory^7,8^, but this approach is limited to GPCRs for which sufficient structural and sequence data are available. Alternatively, experimental evolution, which combines random mutagenesis and high-throughput selection of novel specificities, can identify SDPs without prior knowledge of GPCR structure or sequence homology.

We sought to further our understanding of how ligand specificity can change by focusing on the pheromone receptor Ste2 in the yeast *Saccharomyces cerevisiae*. Like other ascomycetes, *S*. *cerevisiae* senses short peptide pheromones from nearby compatible mates through the use of GPCRs. Upward of 70 yeast species are known to harbor unique receptor-pheromone pairs that act as major determinants of sexual compatibility^9,10^. This impressive diversity has common evolutionary roots, providing fertile grounds for studying how ligand discrimination arose with each speciation event. Furthermore, the *S*. *cerevisiae* lineage is thought to have originated through a rare instance of interspecies hybridization^11^, a scenario which could lead to a hybrid with a “mismatched” receptor and pheromone pair. However, how the GPCR becomes responsive to the new ligand but irresponsive to its former ligand remains unclear. We hypothesized that changes in ligand specificity may not require extensive remodeling of the receptor’s binding pocket, but may instead proceed from changes in signal regulation and/or small differences in the receptor’s structure.

**Figure 1:**
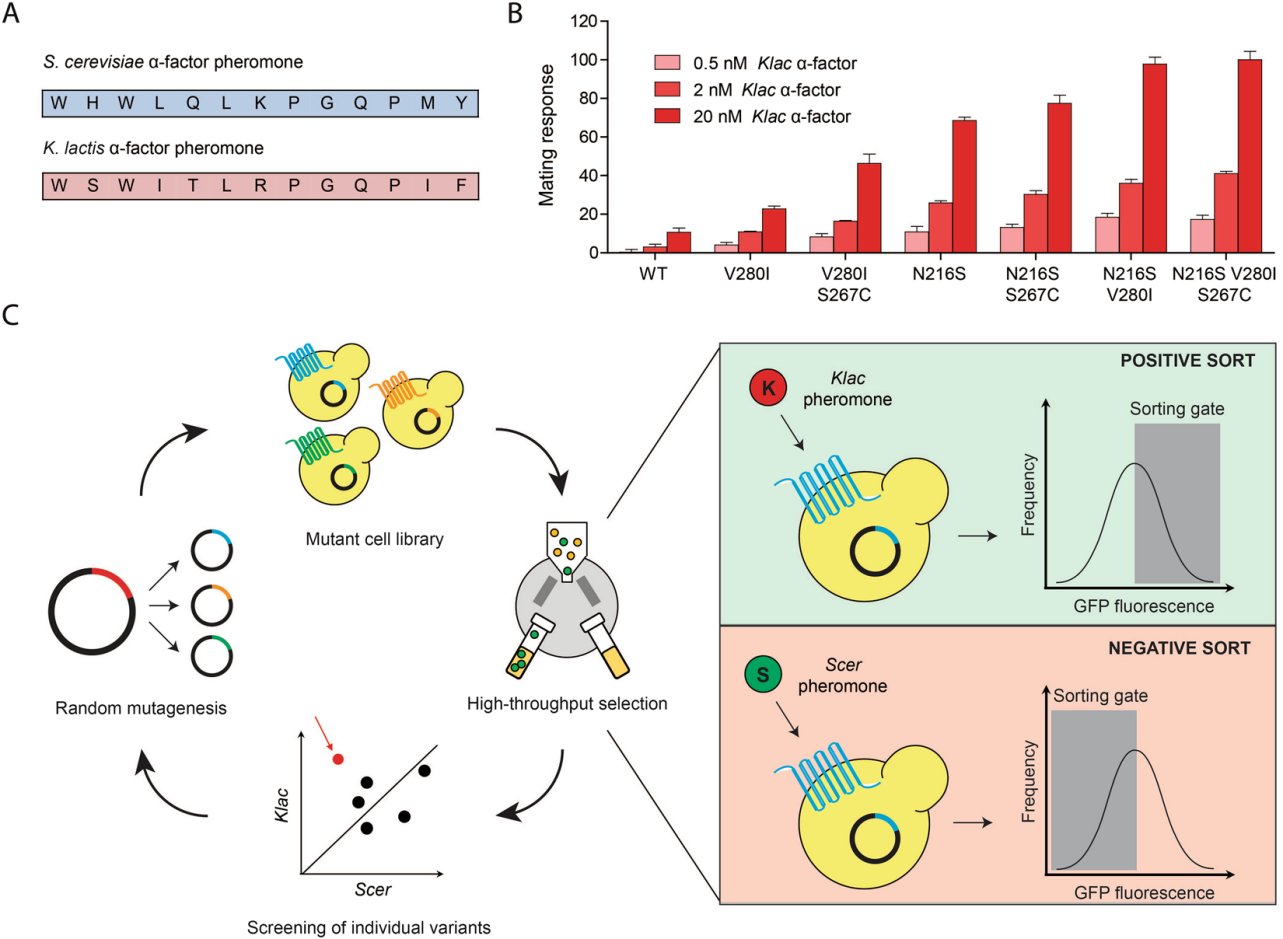
The directed evolution of a broad-specificity variant of the GPCR Ste2 to obtain a ligand-discriminating variant. (**A**) The primary structures of the *S*. *cerevisiae* and *K*. *lactis* peptide pheromones. (**B**) Mating response of cells expressing various broad-specificity Ste2 mutants in the presence of different concentrations of *Klac* pheromone. We selected Ste2 N216S V280I as the starting point of our directed evolution experiment due to its high sensitivity and its small number of mutations. Error bars represent the standard error of the mean (s.e.m.), (**C**) Schematic representation of the iterative process underlying directed evolution. An initial Ste2 ORF is amplified using error-prone polymerase and the resulting amplimers are cloned in a plasmid vector. Yeast cells expressing the mutant Ste2 library are sorted based on their strong response to *Klac* α-factor and their weak response to *Scer* α-factor, as measured from a GFP reporter of mating. Sorted candidates are then screened to confirm their response profile. Further rounds of random mutagenesis and selection can be performed on promising Ste2 variants.

We previously used an experimental evolution approach to understand how *S*. *cerevisiae* (hereafter abbreviated *Scer*) can become responsive to the α-factor pheromone peptide of a related yeast species, *Kluyveromyces lactis* (*Klac*)^12^. This foreign ligand triggers only a weak response with wild-type (WT) Ste2 despite its similar amino acid sequence to *Scer* α-factor (Fig. 1A), but we found that single point mutations in the receptor could increase pheromone potency to high levels. Several Ste2 variants achieved this without displaying greater binding affinity for *Klac* α-factor, but had instead facilitated pathway activation by shedding a regulatory region. However, in all cases, the new Ste2 variants still responded strongly to the native *Scer* pheromone. Though our work revealed different ways that Ste2 can respond to a foreign ligand, it left open-ended the question of how ligand discrimination arises from a broad-specificity receptor.

Here, we aimed to gain further insights on the evolution of ligand discrimination in Ste2. For this, we used directed evolution in order to obtain a variant which exhibits a strong preference for *Klac* pheromone. We investigated the effects of the mutations selected on sensitivity and binding affinity. Remarkably, we found that binding affinity did not play a significant role in discrimination against the native pheromone for our variant. Instead, the ability of each ligand to induce receptor activation, i.e. their efficacy, appears to have become the major determinant of specificity.

## Results

### Iterative rounds of mutations and selection yield *Klac* pheromone-preferring variants

In order to measure the mating response of yeast cells to either *Scer* or *Klac* α-factor, we used a reporter strain in which the mating-inducible promoter of the gene *FUS1* drives the expression of green fluorescent protein (GFP)^13^. Using this strain, we previously uncovered several broad-specificity Ste2 variants that conferred responsiveness to *Klac* pheromone^12^. In order to select a starting point for the directed evolution of a ligand-discriminating variant, we focused on the high-affinity mutations N216S, V280I and S267C since we sought a strong interaction with both pheromones as a baseline. We also sought to maximize sensitivity to *Klac* α-factor in our initial variant since we anticipated that suppression of the response to *Scer* α-factor might not be entirely ligand-specific. We previously showed that N216S or V280I alone is sufficient to enable a strong response to *Klac* pheromone without impairing receptor expression and trafficking, while S267C has a milder effect on sensitivity. Here, we found that combining N216S and V280I greatly increased sensitivity to *Klac* α-factor, while a triple mutant including S267C was not significantly improved (Fig. 1B). As we were concerned about the effect of adding unnecessary mutations on overall protein stability, we decided to use Ste2 N216S V280I as our initial variant from which we might obtain a *Klac* pheromone-specific receptor.

**Figure 2:**
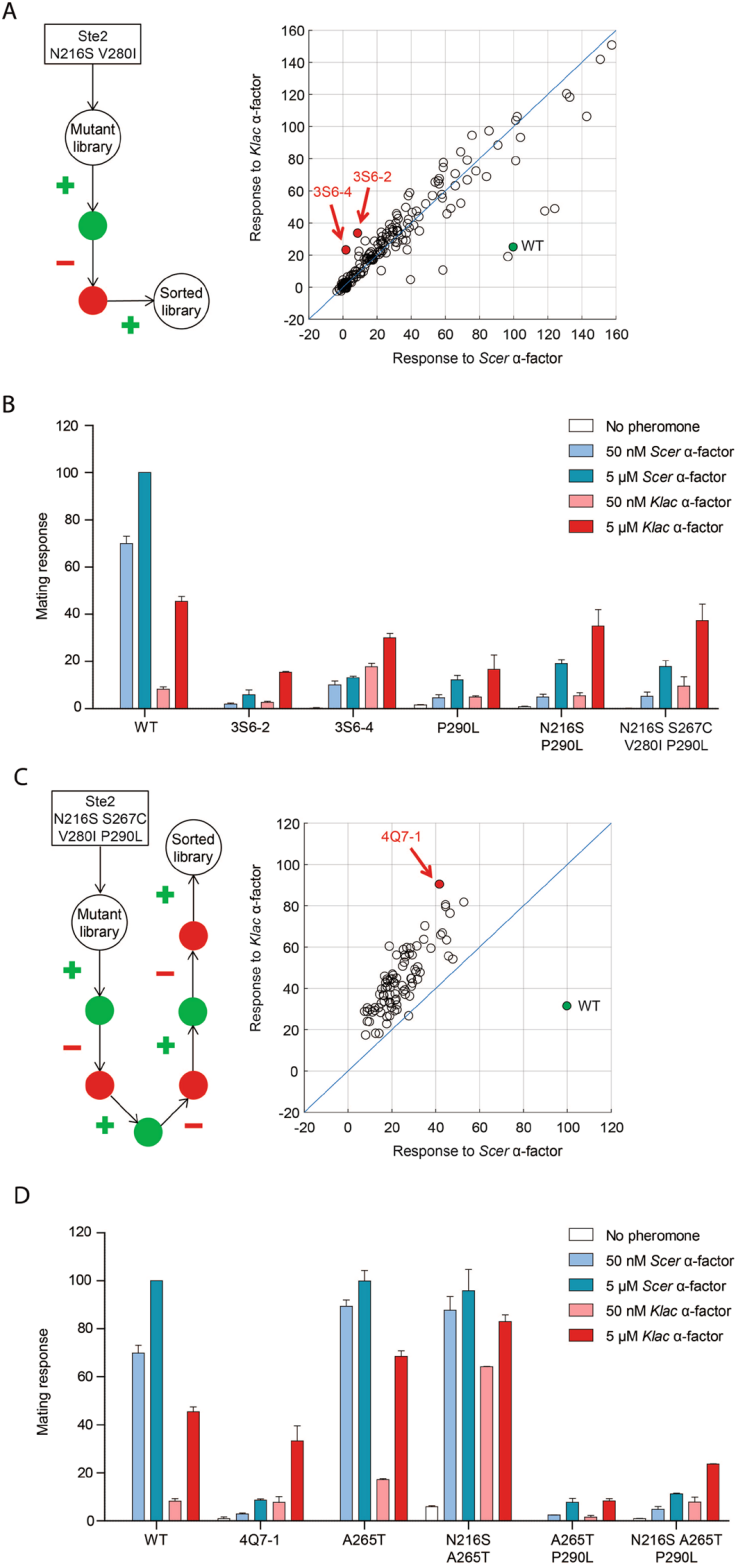
Identifying mutations that confer a new ligand specificity from a sorted library of Ste2 variants. (**A**) Screening of promising candidates following three rounds of sorting from a library of random mutants based on Ste2 N216S V280I. The mating response was measured for each candidate following treatment with 1 µM of each pheromone. The response values are shown in a scatter plot where the diagonal indicates equal *Klac* and *Scer* pheromone-induced activation. Preference for *Klac* pheromone was confirmed in two variants, 3S6-2 and 3S6-4. (**B**) The Ste2 mutation P290L confers *Klac* pheromone preference in the presence of N216S and other sensitizing mutations, but also reduces overall receptor activity. The mating response of each Ste2 variant to either pheromone is shown in bar plots derived from duplicate experiments. Error bars represent the standard error of the mean (s.e.m.), (**C**) Screening of promising candidates following seven rounds of sorting from a library of random mutants based on Ste2 N216S S267C V280I P290L. The mating response is shown in a scatter plot similar to the one in (**A**). Preference for *Klac* pheromone was confirmed in variant 4Q7-1. (**D**) The Ste2 mutation A265T further suppresses the response to *Scer* pheromone in the presence of N216S and P290L. The mating response of each Ste2 variant is shown in bar plots similar to the one in (**B**).

Directed evolution consists of an iterative process combining multiple rounds of mutagenesis and selection (Fig. 1C). We introduced random mutations in the entire open reading frame (ORF) of Ste2 N216S V280I and generated a library of ~100 000 unique mutants. This library was transformed in a yeast Δ*ste2* GFP reporter strain, yielding ~150 000 colony-forming units. Following pheromone treatment, we used fluorescence-activated cell sorting (FACS) to select variants according to their GFP levels. In order to obtain variants with a preference for *Klac* pheromone, we performed a series of positive (+) and negative (−) sorts. This dual selection approach has been used successfully to obtain discriminating receptors in the past^14,15^. In a + sort, cells were treated with *Klac* α-factor and those expressing high levels of GFP were selected. In a – sort, cells were treated with *Scer* α-factor and those expressing little or no GFP were selected. We experimented with various combinations of + and – sorts and found that alternating the two worked best (data not shown). Following a + - + selection routine, we screened 184 variants individually for their ability to respond to either pheromone (Fig. 2A). Among these, we identified two variants, 3S6-2 and 3S6-4, with a small preference for *Klac* α-factor over the native pheromone. Strikingly, both variants shared a common non-seed mutation: P290L.

We confirmed that P290L was responsible for the inverted specificity of 3S6-2 and 3S6-4 by introducing it in a WT or N216S background (Fig. 2B). Interestingly, we found that P290L did not invert specificity on its own. Instead, the sensitizing mutation N216S was necessary to observe a preference for *Klac* α-factor, although this was difficult to ascertain due to the weakness of overall signaling in Ste2 P290L. The addition of P290L was accompanied by reduced surface expression levels (data not shown). Combining P290L to the sensitizing mutations N216S, S267C and V280I enhanced overall signaling.

In order to identify additional mutations promoting *Klac* pheromone specificity, we performed a second round of mutagenesis and selection on the quadruple mutant Ste2 N216S S267C V280I P290L. This time, we did not find a suitable variant until the seventh round of sorting, one that followed a + - + - + - + selection pattern. During our initial screen of this sorted library, we identified variant 4Q7-1 which showed a high *Klac*-to-*Scer* response ratio (Fig. 2C). However, following plasmid extraction and transformation into new cells, this variant exhibited a much lower ratio. We suspect that this discrepancy was due to a cell-specific genomic mutation enhancing overall mating pathway response. Despite this, we found that one of the mutations in this variant, A265T, improved *Klac* α-factor sensitivity dramatically but did not invert ligand specificity on its own or with N216S (Fig. 2D). Instead, this mutation enhanced selectivity slightly when combined with both N216S and P290L by suppressing the response to *Scer* α-factor.

### A combination of ligand-discriminating and sensitizing mutations results in the optimal variant

**Figure 3:**
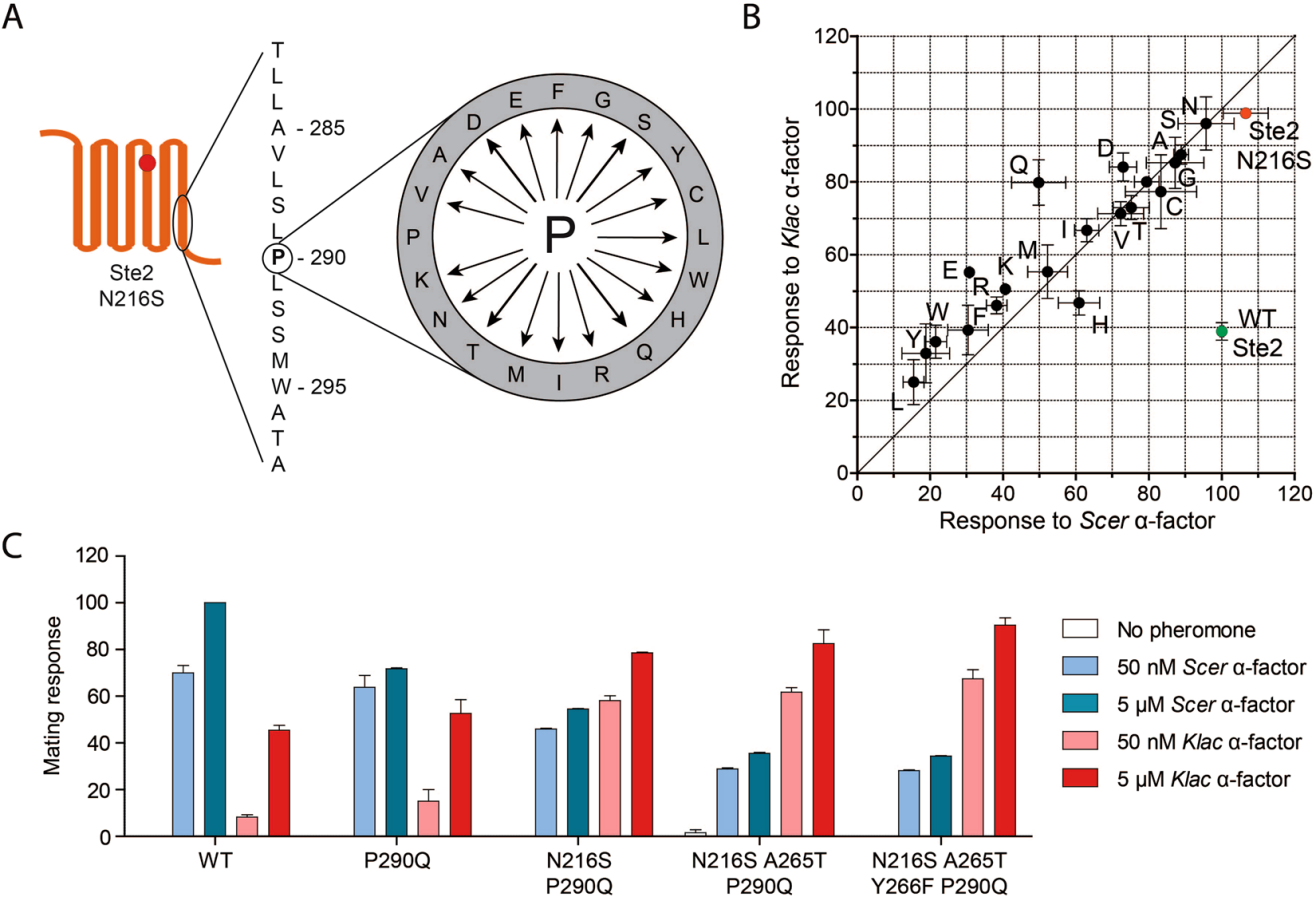
Site saturation mutagenesis identifies mutation P290Q as a superior alternative to P290L. (**A**) The amino acid residue 290, a proline in WT Ste2, was changed to every other natural amino acid in a Ste2 N216S background by using site-directed mutagenesis. (**B**) Mutations at site P290 lead to a diverse set of response profiles. The mating response was measured for each variant following treatment with 5 µM of each pheromone. The response values are shown in a scatter plot where the diagonal indicates equal *Klac* and *Scer* pheromone-induced activation. Variants are labeled according to the amino acid present at site P290, where variant “P” represents Ste2 N216S and “WT” represents WT Ste2. (**C**) The mutation P290Q can be combined with other mutations to yield a receptor with inverted specificity. The mating response of each Ste2 variant to either pheromone is shown in bar plots derived from duplicate experiments. Error bars represent the standard error of the mean (s.e.m.).

Although random mutagenesis and selection successfully identified P290L as crucial for enabling a preference for *Klac* pheromone, its detrimental effect on overall signaling was problematic. In order to obtain a better variant, we reasoned that the site P290 was likely more important than the mutation itself, and so we proceeded to test all other amino acid substitutions at this site, an approach known as site saturation mutagenesis (Fig. 3A). These substitutions were introduced in a Ste2 N216S background due to the apparent cooperative effect of this mutation with P290L. The resulting variants were then assayed for their sensitivity to saturating concentrations of either *Klac* or *Scer* pheromones and compared to WT Ste2 (Fig. 3B). We observed a broad range of phenotypes in P290 mutants, suggesting that this site can tune both receptor activity and specificity. Several substitutions, such as those involving aromatic and charged residues, resulted in an inversion of specificity but also a lower overall response. In that regard, P290Q proved to be a superior alternative to P290L. In fact, no mutation was as detrimental to overall receptor activity as P290L. A non-saturating concentration of pheromone yielded broadly similar results (Supplementary Figure 1).

**Figure 4:**
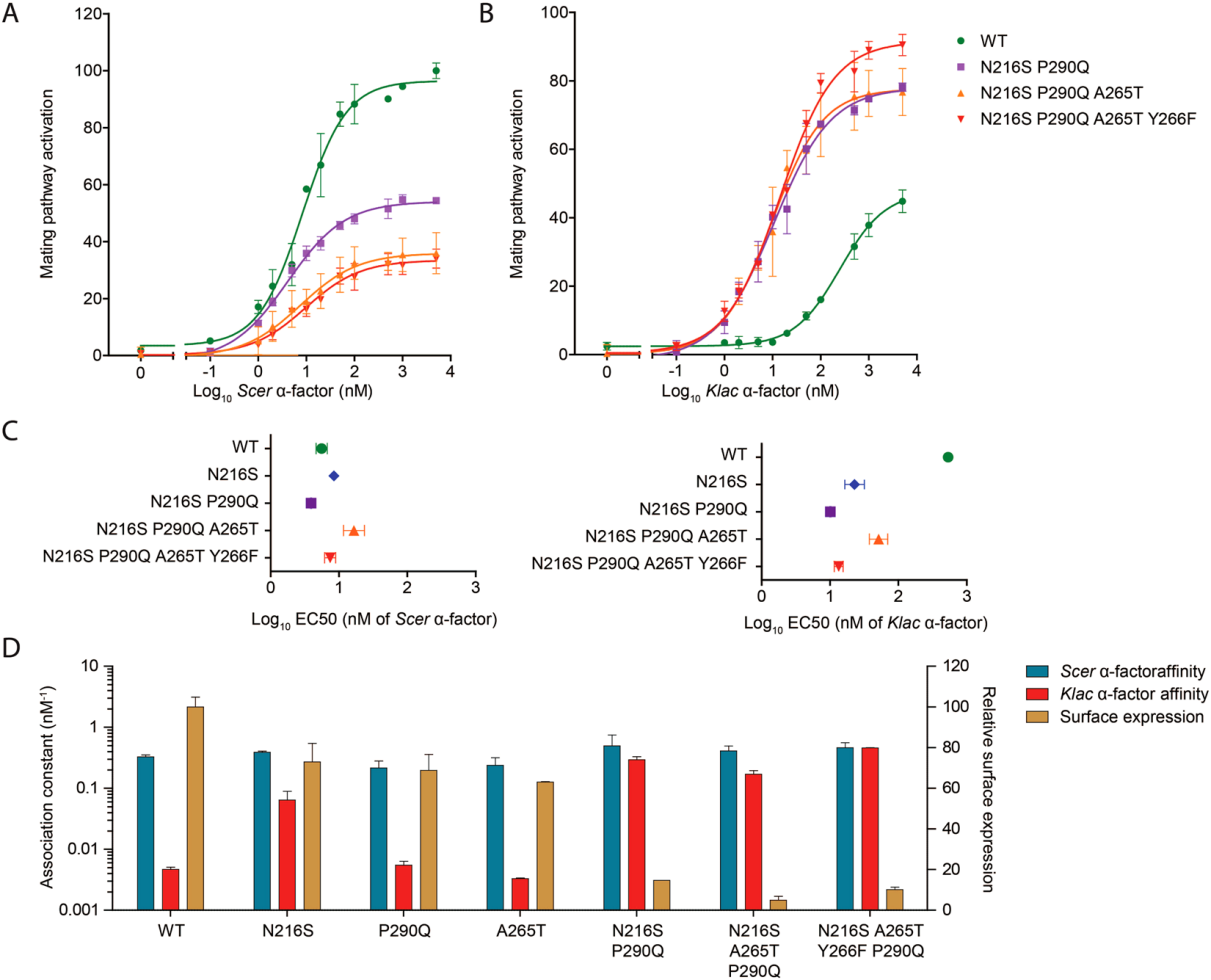
The Ste2 variant harboring mutations N216S, P290Q, A265T and Y266F discriminates between *Scer* and *Klac* pheromones through differences in ligand efficacy. (**A,B**) Dose-response curves showing the effects of adding sensitizing mutations (N216S and Y266F) and discriminating mutations (P290Q and A265T) to the mating response profile of cells treated with either *Scer* or *Klac* pheromones. (**C**) Mating response sensitivity for the Ste2 variants assayed in (**A**) and (**B**). EC50 values were derived from duplicate dose-response experiments. (**D**) Binding constants and surface expression levels of Ste2 variants harboring one or multiple mutations identified in this study. The binding affinity for *Scer* α-factor and the levels of surface expression were obtained from duplicate saturation binding assays. The binding affinity for *Klac* α-factor was obtained from duplicate competition binding assays. All variants could bind to *Scer* pheromone with similar affinity (nonparametric Kruskal-Wallis test), suggesting that this was not the basis of ligand discrimination observed at the mating response level. Error bars represent the standard error of the mean (s.e.m.).

On its own, mutation P290Q appears to act largely by suppressing the receptor’s response to *Scer* pheromone (Fig. 3C). This effect is greater in the presence of N216S, suggesting that the latter mutation is not simply sensitizing. Rather, mutations at positions 216 and 290 are epistatic. Although Ste2 N216S P290Q is able to clearly discriminate between native and foreign pheromones, we attempted to improve its low activity without impairing specificity. First, we found that adding mutation A265T resulted in even greater suppression of the *Scer* α-factor response specifically. Second, we were able to further increase the response to *Klac* α-factor specifically with the sensitizing mutation Y266F^12,16^. As we were unsure whether A265T and Y266F contributed usefully to our desired phenotype, we performed a more detailed analysis of each variant’s response profile by measuring dose-response curves and EC50 values. This revealed that A265T reduced the maximum response to *Scer* α-factor when added to Ste2 N216S P290Q (Fig. 4A), while the response to *Klac* α-factor was unaffected (Fig. 4B). Conversely, Y266F had the opposite effect on the maximum response to each pheromone when added to Ste2 N216S P290Q A265T. Likewise, EC50 values showed that Y266F re-balanced the desensitizing effect of A265T (Fig. 4C). Thus, both mutations were beneficial and the resulting Ste2 variant harboring mutations N216S P290Q, A265T and Y266F, hereafter abbreviated to Ste2 K-Switch, displayed a full inversion of specificity for the *Klac* and *Scer* pheromones compared to the WT.

### Differences in binding affinity are not necessary to acquire a novel specificity

GPCR activation is a multi-step process involving at the very least a ligand binding step that is distinct from subsequent conformational changes in receptor structure. We wondered if the ability of Ste2 K-Switch to discriminate between the *Scer* and *Klac* pheromones could be explained by their relative binding affinity. Through the use of a fluorescently-labelled pheromone, we measured the binding affinity and surface expression levels of our Ste2 variants and made several observations. First, the affinity for *Scer* α-factor was consistently high (Fig. 4D) and there was no significant difference between the variants analyzed (one-way ANOVA). This suggested that P290Q and A265T do not cause weaker *Scer* pheromone binding, but rather that they alter the ability of each pheromone to activate the receptor, a property known as efficacy. Second, affinity to *Klac* α-factor was much improved by the mutation N216S, and only marginally by P290Q. Third, and perhaps most unsurprisingly, we found that variants harboring multiple mutations showed lower surface expression, most likely due to destabilizing effects. However, the strong mating response observed in these suggested that their reduced receptor levels were still adequate. We also confirmed that overexpression of Ste2 K-Switch did not affect its response profile (Supplementary Figure 2).

### *Klac* and *Scer* pheromone variants reveal that positions 2 and 12 of α-factor determine receptor specificity

**Figure 5:**
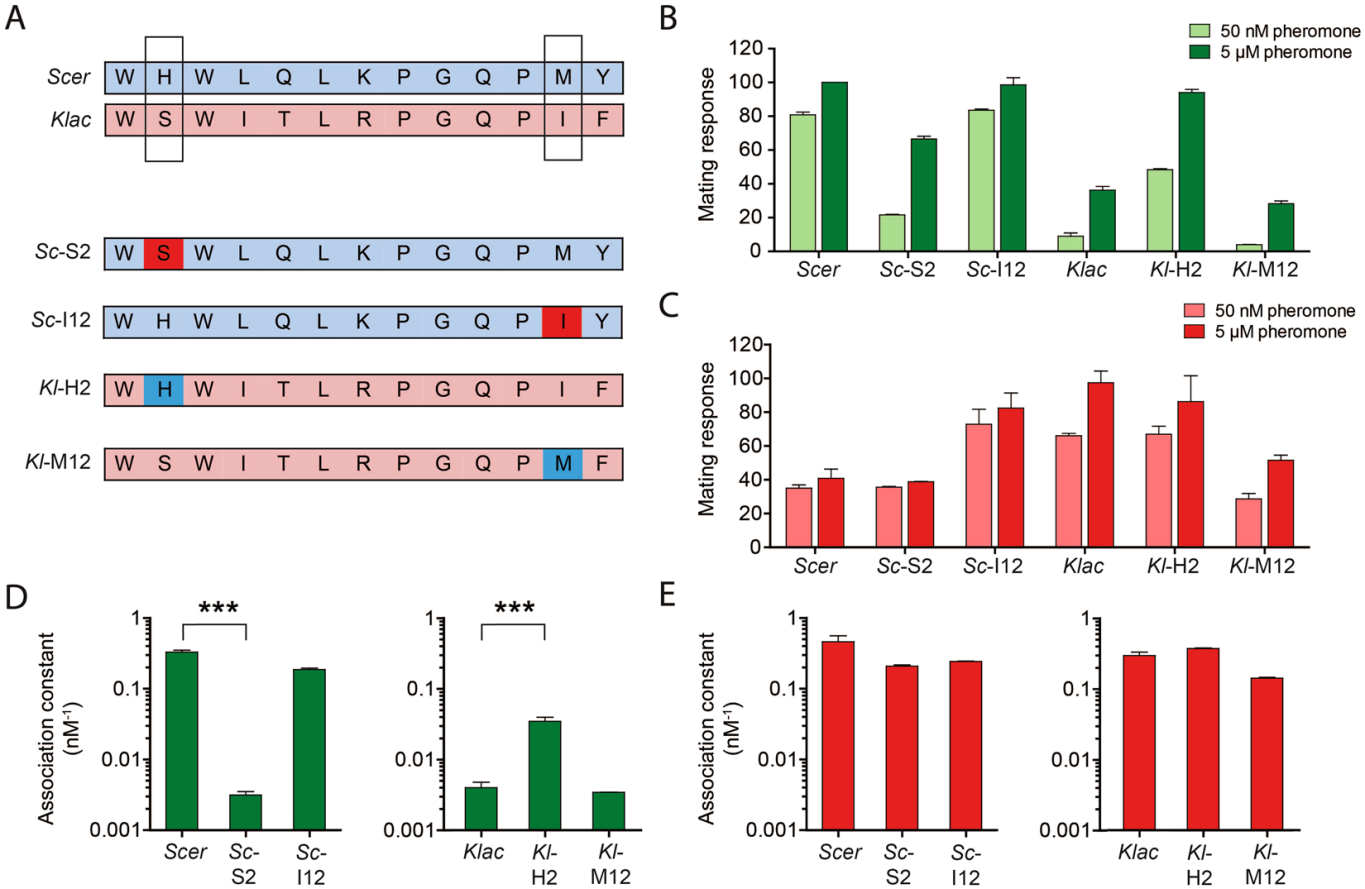
Position 2 and position 12 of the peptide pheromones determine the ligand specificity of WT Ste2 and Ste2 K-Switch. (**A**) The primary structures of the main pheromone variants used in this study. The WT pheromones are labeled *Scer* and *Klac* and positions 2 and 12 are boxed. The other pheromone variants harbor single amino acid substitutions with respect to the WT sequences, which are highlighted by their different colours, and are named accordingly. (**B**,**C**) The mating response of WT Ste2 (**B**) and Ste2 K-Switch (**C**) to each pheromone variant is shown in bar plots derived from duplicate experiments. Position 2 controls the WT response, while position 12 controls the response in the *Klac* pheromone-specific variant. (**D**,**E**) Binding constants of WT Ste2 (**D**) and Ste2 K-Switch (**E**) to each pheromone variant. Binding affinity values were obtained from at least two competition binding assays. Asterisks denote statistically significant differences between values (***P < 0.001) based on a Bonferroni-corrected multiple comparison test. WT can distinguish between *Scer* and *Klac* based on binding affinity and this is strongly linked to the amino acid at position 2 of each peptide, while Ste2 K-Switch does not clearly distinguish between any pheromone variant through binding affinity. Error bars represent the standard error of the mean (s.e.m.).

The pheromone peptides of *Klac* and *Scer* are both 13-amino acid long and share a similar sequence (Fig. 5A). We sought to identify the amino acids responsible for the opposite specificities of WT Ste2 and Ste2 K-Switch by using pheromone variants focusing on the most significant biochemical differences between the two peptides, positions 2, 5 and 12. Interestingly, we found that changing position 2 of the *Scer* pheromone to the *Klac* equivalent (His to Ser) decreased its efficacy with WT Ste2 while the opposite effect was observed when using a *Klac* peptide where position 2 was changed to the *Scer* equivalent (Ser to His) (Fig. 5B). For Ste2 K-Switch, changing position 12 of *Scer* pheromone to the *Klac* equivalent (Met to Ile) enhanced the response dramatically, while the reverse substitution (Ile to Met) in the *Klac* pheromone reduced its activity (Fig. 5C). Remarkably, position 12 substitutions did not affect WT signaling, while the same was true for position 2 and Ste2 K-Switch. Finally, substitutions at position 5 had no effect alone, or in combination with the other substitutions (Supplementary Figure 3). These results suggest that position 2 and 12 are each important for ligand discrimination in WT Ste2 and Ste2 K-Switch respectively.

Binding assays revealed that changes at positions 2 and 12 contribute to ligand discrimination in distinct ways. Substitutions at position 2 affected binding affinity to WT Ste2 by either decreasing it (Ser) or by increasing it (His) significantly (Fig. 5D). On the other hand, changing position 2 or position 12 had little effect on the binding affinity to K-Switch (Fig. 5E). This further supports the notion that Ste2 K-Switch does not distinguish between *Klac* and *Scer* on the basis of binding affinity, but rather does so through differences in efficacy.

## Discussion

In the present study, we used a laboratory evolution approach to completely invert the specificity of a GPCR relative to its WT state. The Ste2 mating receptor contributes to the sexual isolation of the yeast *Scer* through its incompatibility with the pheromone of the related species *Klac*. Through a combination of random mutagenesis, high-throughput selection and site-saturation mutagenesis, we identified a set of four mutations that render Ste2 fully responsive to *Klac* α-factor but also incompatible with its original cognate ligand. Importantly, we have found that a GPCR can evolve to discriminate between related ligands in at least two distinct ways. Unlike WT Ste2, the *Klac*-specific Ste2 variant does not distinguish between pheromones based on binding affinity. Rather, the new mutations specifically reduce the ability of *Scer* α-factor to trigger pathway signaling, i.e. its efficacy. The difference in ligand recognition between the WT and the mutant is linked to two amino acid residues on the peptide pheromones. These results show that a novel GPCR-ligand specificity can arise without changes in binding affinity, while the efficacy-suppressing mutations identified here point to novel SDPs in the yeast mating receptor.

**Figure 6:**
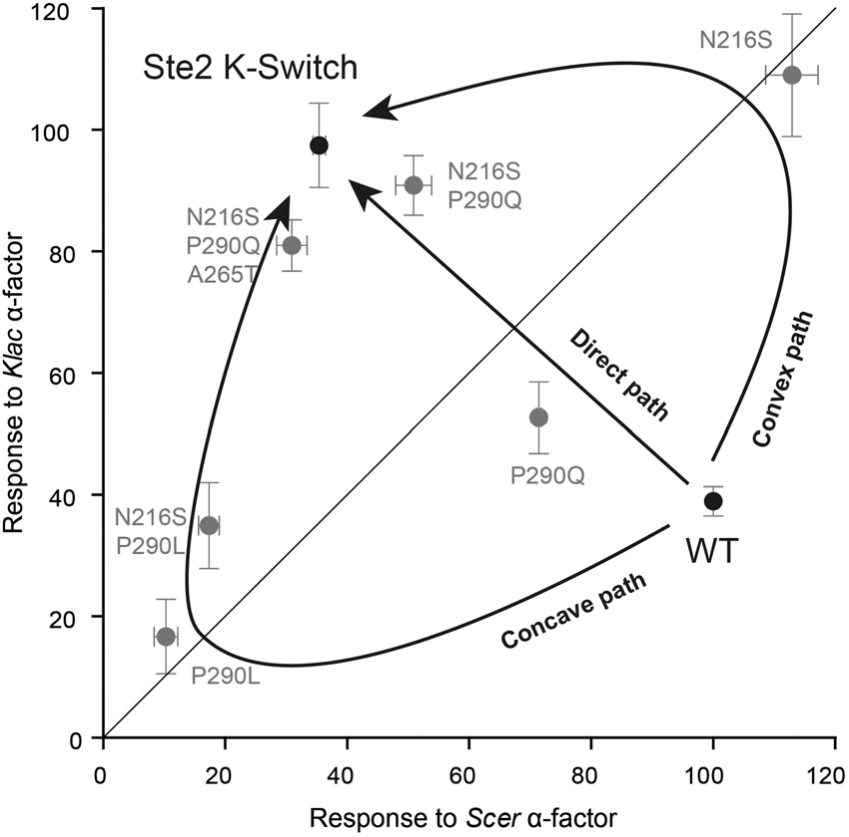
Ste2 can evolve a new ligand specificity via different evolutionary paths. A scatter plot of the relative mating response of the Ste2 variants used in this study reveals the Ste2 specificity landscape. The different ways in which an inversion of specificity can occur are indicated by arrows and labeled by the shape of their trajectory. Our study uncovered all three possible evolutionary intermediates: broadly-activated (N216S, convex), broadly inactivated (P290L, concave) and balanced specificity (P290Q, direct). Error bars represent the standard error of the mean (s.e.m.).

Laboratory evolution has proven to be a powerful approach for visualizing the evolutionary path of proteins in real-time^12,17,18^. Studies using this method have shown that in order to alter the specificity of a protein, be it enzyme or receptor, it is preferable to go through a generalist stage where it can promiscuously interact with both the new and the old substrate or ligand^14,15,19–22^. This can then be followed by a re-specialization step, where the old activity is largely abolished. This evolutionary path is sometimes called “convex”, due to its shape on a map of relative activity and is characterized by a weak negative trade-off between each specificity^23^. The alternatives, where a protein switches specificity instantly (direct or straight path) or where it goes through an inactive stage (concave path), are thought to be less likely due to the need to maintain a strong activity for at least one substrate/ligand. In agreement with this, ancestral gene resurrection studies have found that promiscuity is a recurrent feature of ancestral proteins, supporting the view that the convex path is favored in real evolution^24,25^. In our own experiment, we uncovered convex, concave and direct trajectories (Fig. 6). The variant Ste2 N216S is a clear generalist intermediate, and so its occurrence is part of a convex path. However, mutations at site P290 can lead to different routes. P290L makes the path to a specialist concave, while P290Q can either continue on the convex route to specificity, or form a direct route, depending on whether it occurs before or after N216S. Our strict selection strategy involving non-overlapping sorting gates may explain why we found P290L first, but it is likely that P290Q would have been preferable in nature due to its compromise between functionality and specificity. This highlights the value of combining multiple mutagenesis methods: although random mutations were necessary to identify a functionally significant site with little prior knowledge of Ste2′s structure (P290), it was saturation mutagenesis that enabled us to find the optimal amino acid residue at this site (Q).

Pioneering work by Marsh *et al*. on Ste2 and the pheromone of *Saccharomyces kluyveri* revealed that promiscuity can be achieved via point mutations or hybridization of the *Scer* and *S*. *kluyveri* receptors^26,27^. A primary aim of our study was to determine how ligand discrimination can arise in such promiscuous GPCRs. We hypothesized that although the ligand-receptor interface would play a major role, it would not be limited to changes in binding affinity. We reasoned that the binding pocket of a GPCR, which involves interactions with multiple helices, would be unlikely to undergo significant re-modeling in order to exclude its native ligand. Instead, multiple studies have shown that a new specificity does not lie very far; a small number of mutations is sufficient^15,18^. Our findings support this view, as N216S and P290Q epistatically^17^ induce a reasonable level of ligand discrimination and activity. Furthermore, we found that WT Ste2 and Ste2 K-Switch distinguish between pheromones in very different ways. While N216S rendered the Ste2 binding pocket permissive to both *Klac* and *Scer* pheromones, its combination with P290Q and A265T did not restrict *Scer* α-factor binding, but instead reduced its efficacy specifically. This stands in contrast to WT Ste2, which relies at least partly on binding affinity to ensure a weak response to related pheromones. Interestingly, this implies that *Scer* α-factor became a competitive antagonist for *Klac* α-factor in our variant. In nature, unlike in our experiment, there would likely be additional selection pressure against antagonism which would favor geographic isolation, since competition for receptors would weaken signaling wherever *Scer* cells are present^28,29^. While previous work in GPCRs demonstrated that ligand efficacy is an important contributor to specificity alongside binding affinity^8,30^, our results indicate that it can be a sole contributor under certain conditions of selection. However, unlike our variant, the specificity of WT Ste2 is the result of millions of years of sexual isolation and receptor-pheromone co-evolution in a diverse environment. Advances in library design, high-throughput methods of selection and *in silico* evolution could eventually be used to reveal the broader range of mutations that accompany multi-ligand discrimination.

Due to a lack of comprehensive 3D structural data for Ste2 and other fungal GPCRs, we can only speculate on what drives the suppression of *Scer* efficacy among our variants. For N216S, past work showed that another mutation at site, N216D, can counter the effects of the loss of function mutations Y266C and F204S, each of which affects either ligand binding or receptor activation, making this site relevant to both processes^31^. Likewise, A265T is adjacent to Y266, a crucial residue in Ste2 function which is thought to interact with the pheromone’s N-terminus and take part in the subsequent conformational change that accompanies receptor activation^32^. But the most important mutation that we found, P290Q, is perhaps also the most puzzling. Prolines are known to interrupt helical structures, and a NMR analysis of Ste2′s seventh transmembrane domain suggests that P290 is no exception^33^. Prolines are well-tolerated throughout the seventh transmembrane domain of Ste2, suggesting that this helix does not need to be continuous for proper signaling^34^. Our results support this (e.g. P290N), but many other substitutions at this site do change function dramatically by inverting specificity. This is especially surprising when considering the apparent lack of involvement of P290 in *Scer* pheromone binding or receptor activation as probed in past studies^31,35–37^. Our work suggests that P290 plays a significant role in establishing ligand discrimination, even if it does not contact the pheromones directly. Furthermore, this role appears to be linked to positions 2 and 12 of the pheromones. Specifically, we found that position 2 substitutions affected binding and efficacy with the WT receptor, while position 12 substitutions affected only efficacy with Ste2 K-Switch. The precise function of each of α-factor’s amino acid residues has been probed using a variety of peptide analogs and mutants, with some studies suggesting that the pheromone harbors distinct signaling and binding domains at the N-terminus and C-terminus respectively^32^, while other studies showed significant overlap between these two roles^38,39^. Our results support the latter view, but the divergence in the field highlights the need for further studies using systematic substitutions beyond alanine-scanning, as was done recently by Stainbrook *et al*.^40^, as well as exploring the diversity of yeast pheromones and their ability to create sexual barriers between related species.

In conclusion, our work demonstrates that the evolution of a novel GPCR ligand specificity can proceed from a promiscuous variant through changes in ligand efficacy rather than binding affinity. Furthermore, our work establishes a novel relationship between the proline of Ste2′s seventh transmembrane domain and position 2 and 12 of bound pheromones. These results may be especially relevant for GPCRs in higher eukaryotes with peptide ligands (chemokine receptors, opioid receptors, neuropeptide receptors, etc.) or peptide antagonists. Lastly, while we benefited from the relatedness of *Scer* and *Klac* mating apparatus, the ascomycete family includes many more species with additional receptor-pheromone homologs, providing further opportunities to improve our understanding of peptide discrimination and ligand-receptor co-evolution in GPCRs^41,42^.

## Methods

### Strains and growth conditions

The *S*. *cerevisiae* strains used in this study were derived from strain W303 and are listed in Supplementary Table 1. For all assays, yeast strains were first transformed with plasmids by the lithium acetate / polyethylene glycol method^43^ and grown on selective synthetic defined (SD) plates. Transformed colonies were picked and grown overnight in selective liquid SD medium in a 30 °C shaking incubator. Cultures were then diluted to an OD = 0.1 and grown to exponential phase (OD = 0.5 to 0.8) before all treatments. Liquid SD medium was prepared from 6.74 g/L of yeast nitrogen base with ammonium sulfate without amino acids (BioShop), 1 g/L of the appropriate amino acid drop-out mix (BioShop) and 2% v/v glucose. Solid SD medium was prepared by adding 20 g/L of agar to liquid SD.

### Plasmid propagation and construction

The plasmids and oligonucleotides used in this study are listed in Supplementary Tables 2 and 3 respectively. Plasmid propagation in *Escherichia coli* DH5α was done with Luria Bertani (LB) medium supplemented with 50 µg/mL carbenicillin, and cultures were grown at 37 °C. For plasmid construction, promoters were amplified from yeast genomic DNA (Invitrogen) and cloned at the PspOMI and XhoI sites in a pRS313 backbone. Ste2 ORFs were cloned at AarI sites.

### Peptide preparations

Peptides were synthesized externally (Biomatik) to at least 95% purity. Peptides were dissolved in dimethyl sulfoxide (DMSO) to a stock concentration of 10 mM.

### Mating pathway activation assays

Transformed RB001 cells from two independent colonies were treated with one or a series of concentrations of pheromone and placed in a 30 °C shaking incubator. After 3 hours, protein synthesis was inhibited by treating cells with 10 µg/mL cycloheximide. The intensity of fluorescence in the 525/50 nm range was measured by analytical flow cytometry with a MACSQuant VYB (Miltenyi Biotec) equipped with a 588 nm laser. Cells without receptors were used to subtract basal cell fluorescence from other samples to reveal the net GFP fluorescence. For dose-response assays, the data were fitted with the “log(agonist) vs. response – Variable slope (four parameters)” model in Prism (GraphPad). All experiments included a WT Ste2 control treated with *Scer* α-factor. The fluorescence intensity was normalized to the maximum intensity of the WT control and multiplied by 100. EC50 values represent the mean of two experiments normalized to the WT control of each experiment and multiplied by the mean EC50 of WT Ste2 with *Scer* α-factor. All Ste2 variants were under the control of the STE2 promoter.

### Mutagenesis and selection

For site-directed mutagenesis, Ste2 ORFs were amplified using complementary mutagenic primers and Turbo polymerase (Agilent). For saturation mutagenesis of site 290, codons used are listed in Supplementary Table 4. For random mutagenesis, the full-length open reading frame of Ste2 N216S V280I was amplified by error-prone PCR using the GeneMorph II kit (Agilent). PCR conditions (500 ng of template DNA, 20 cycles) were selected to yield an expected mean mutation rate of 4.0 DNA mutations per ORF^44^. The resulting amplimers were ligated in pRS-p*STE2* and amplified in *E*. *coli* DH5α to generate a mutant library. This library was transformed in the yeast strain RB001. For selection, cells were treated with 5 µM pheromone, incubated for 3 hours in a 30 °C shaker and sonicated briefly. Cell sorting was done in a FACSAria (BD). For intermediate selection rounds, cells were washed in growth medium before being incubated in a 30 °C shaker overnight for sorting the following day. For final selection rounds, cells were plated on solid SD medium. Colonies recovered this way were screened for their ability to activate the mating pathway better in response to either pheromone. Mutant *STE2* plasmids were extracted from the most promising colonies by treating saturated yeast cultures with zymolyase overnight at 4 °C, followed by a standard plasmid miniprep protocol (Qiagen). Plasmids were then amplified in *E*. *coli*, transformed in naïve yeast cells to confirm that they conferred the phenotype, and sequenced.

### Pheromone binding assays

To measure binding affinities and binding site levels, we used the NBD-labelled pheromone binding assay^45^. Transformed RB002 cells were treated with either varying concentrations of NBD-labelled *Scer* α-factor (saturation binding assays) or varying concentrations of *Klac* pheromone mixed with 20 mM NBD-*Scer* (competition binding assays) and left on ice for 10 min. Samples were processed by flow cytometry. For saturation binding, the data were fitted to the “One site – Total and nonspecific binding” model in Prism (GraphPad), and cells without receptors were used to account for non-specific binding. This model uses the equation *Y* = *B*_max_[*L*]/(*K*_d_ + [*L*]) + *N*[*L*] + *Background*, where *Y* is the mean fluorescence, [*L*] is the concentration of labelled ligand and *N* is a proportionality constant for nonspecific binding. For competition binding, the background-subtracted data were fitted to the “One site – Fit Ki” model with the appropriate constraints. This model uses the equation *K*_i_ = *IC*_50_/(1 + [*L*]/*K*_D_). All experiments included a WT control treated with *Scer* α-factor and an empty vector negative control. *K*_D_, *B*_max_ and *K*_i_ values were averaged from at least two experiments and normalized to the WT control of each experiment. In order to ensure a high signal-to-noise ratio even when surface receptor expression was low, we used strong promoters from housekeeping genes. For binding affinity values, all variants were under the control of the *ADH1* promoter with the exception of Ste2 N216S P290Q, Ste2 N216S P290Q A265T and Ste2 K-Switch, which were under the control of the stronger *GPD* promoter due to their weaker expression. For *B*_*max*_ values, all variants were under the control of the *ADH1* promoter.

### Data availability

The data that support the findings of this study are available from the corresponding author upon request.

## Electronic supplementary material

Supplementary Information
