# Supplementary material for: The directed evolution of ligand specificity in a GPCR and the unequal contributions of efficacy and affinity
Source: Sci Rep. 2017 Nov 22;7:16012. doi: 10.1038/s41598-017-16332-2 (PMC5700115; doi:10.1038/s41598-017-16332-2)
Supplement: Supplementary file 1 — Supplementary Information [file 41598_2017_16332_MOESM1_ESM.pdf]

The directed evolution of ligand specificity in a GPCR and

the unequal contributions of efficacy and affinity

Raphaël B. Di Roberto*^, Belinda Chang* and Sergio G. Peisajovich*

*Department of Cell and Systems Biology, University of Toronto

25 Harbord Street, Toronto, ON, M5S 3G5, Canada

^To whom correspondence should be addressed: 25 Harbord Street, Toronto, ON, M5S 3G5, Canada. Phone: +1-416-831-4731, email: raphael.brissetdiroberto@mail.utoronto.ca

SUPPLEMENTARY INFORMATION

SUPPLEMENTARY FIGURES

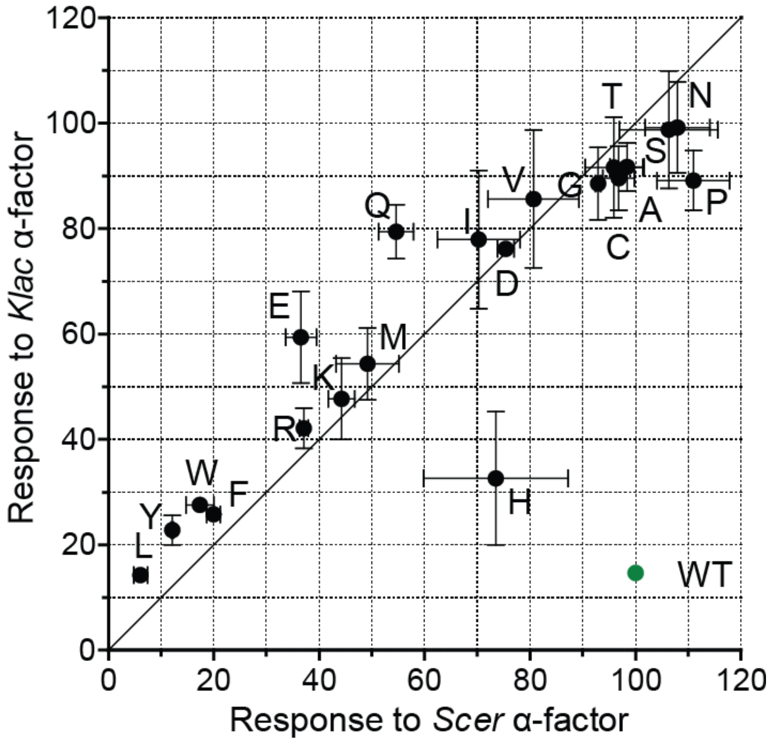

Supplementary Figure 1: The mating response of P290 mutants treated with a non-saturating concentration of pheromone. Mutations at site P290 lead to a diverse set of response profiles when treated with 50 nM of each pheromone. The response values are shown in a scatter plot where the diagonal indicates equal *Klac* and *Scer* pheromone-induced activation. Variants are labeled according to the amino acid present at site P290, where variant "P" represents Ste2 N216S and "WT" represents wild-type Ste2. Error bars represent the standard error of the mean (s.e.m.)

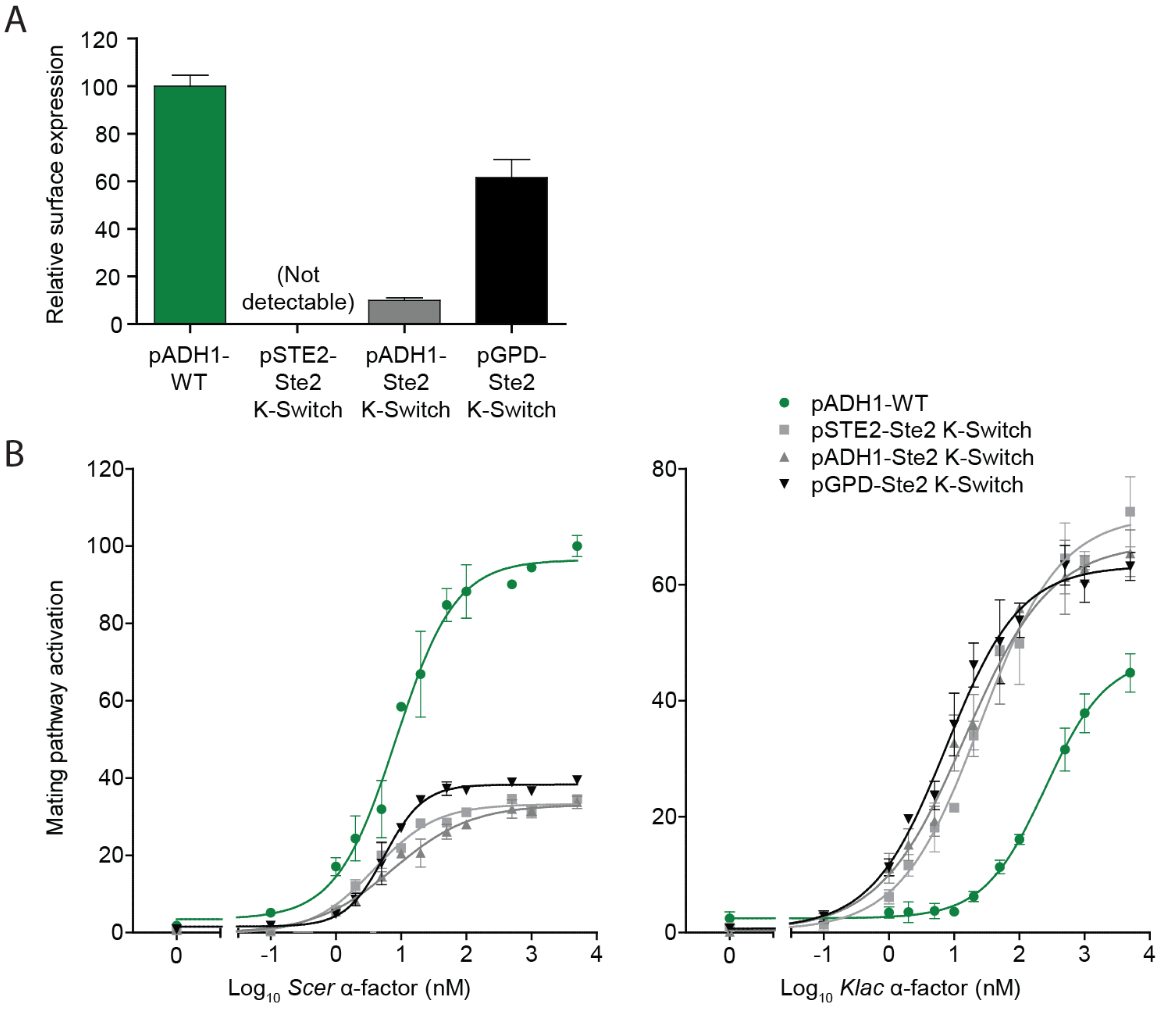

Supplementary Figure 2: The overexpression of Ste2 K-Switch does not affect its response profile. A, Different promoters alter the surface expression levels of Ste2 K-Switch. Surface expression was measured in a saturation binding assay with NBD-*Scer*. The endogenous Ste2 promoter results in undetectable levels of receptor while the promoters of the genes ADH1 and GPD enhance expression to detectable levels. **B,** Different promoters do not alter the response profile of Ste2 K-Switch. Dose-response curves show the negligible effects of enhancing expression for this Ste2 variant. Error bars represent the standard error of the mean (s.e.m.)

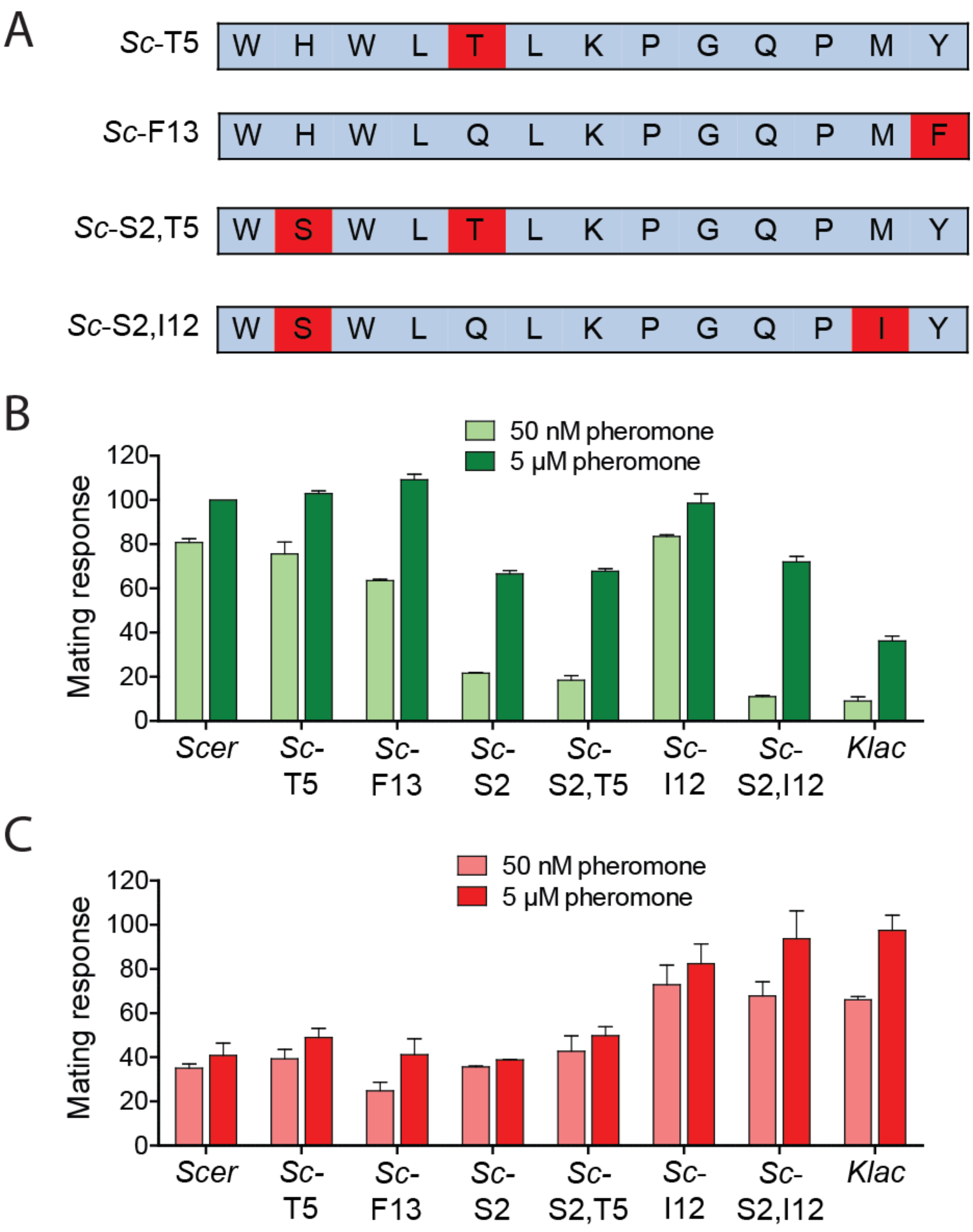

Supplementary Figure 3: Amino acid substitutions at positions 5 and 13 show no effect on ligand discrimination for WT Ste2 and Ste2 K-Switch. A, The primary structures of additional pheromone variants used in this study are shown. These *Scer* α-factor variants harbor single amino acid substitutions with respect to the WT sequences,

which are highlighted by their different colours, and are named accordingly. **B-C,** The mating response of WT Ste2 (B) and Ste2 K-Switch (C) to select pheromone variants is shown in histograms derived from duplicate experiments. The substitution Q5T alone has no effect on efficacy, while combinations with H2S and M12I show no apparent synergy. The conservative substitution Y13F also has no effect. Error bars represent the standard error of the mean (s.e.m.)

SUPPLEMENTARY TABLES

Supplementary Table 1: Yeast strains used in this study

Name	Genotype	Origin
**SΦ992**	*W303 MATa, STE2, SST2, BAR1, FAR1, MFA2, his3, trp1, leu2, ura3*	1
**CB008**	*SΦ992 bar1::NatR, far1Δ*	1
**CB009**	*CB008 mfa2::pFUS1-GFP*	1
**RB001**	*CB009 ste2::TRP1*	2
**RB002**	*CB008 ste2::TRP1*	2

Supplementary Table 2: Plasmids used in this study

Name	Description	Origin
**pRS313**	CEN *HIS3* vector	2
**pRS-P*STE2***	CEN *HIS3 STE2* promoter (P_{STE2})	2
**pRS-P*ADH1***	CEN *HIS3 ADH1* promoter (P_{ADH1})	2
**pRS-P*GPD***	CEN *HIS3 GPD* promoter (P_{GPD})	This study

Supplementary Table 3: Oligonucleotides used in this study

STE2_AarI_5	GCC ATG AAC ACC TGC AAC ACC CTA TGT CTG ATG CGG CTC CTT C	Random mutagenesis of *STE2* ORF with flanking AarI sites
STE2_AarI_3	GTTACAGGCACCTGCAACATCG CTCATAAATTATTATTATC	
SDM_Ste2_280I_5	GAACAGATGTCTTGACTACTATT GCAACATTACTTGCTGTA	Change position 280 in *STE2* to I
SDM_Ste2_280I_3	TACAGCAAGTAATGTTGCAATA GTAGTCAAGACATCTGTTC	
SDM_Ste2_267C_5	TAATATTCATCCTCGCATACTGT TTGAAACCAAACCAGGGA	Change position 267 in *STE2* to C
SDM_Ste2_267C_3	TCCCTGGTTTGGTTTCAAACAGT ATGCGAGGATGAATATTA	
SDM_Ste2_290X_5	TACTTGCTGTATTGTCTTTANNN TTATCATCAATGTGGGCCA	Change position 290 in *STE2* to X (see Supplementary Table 4 for NNN)
SDM_Ste2_290X_3	TGGCCCACATTGATGATAANNN TAAAGACAATACAGCAAGTA	
SDM_Ste2_265T_5	CAT CGA TAA TAT TCA TCC TCG CAT ACA GTT TGA AAC CAA AC	Change position 265 in *STE2* to T
SDM_Ste2_265T_3	GTT TGG TTT CAA ACT GTA TGC GAG GAT GAA TAT TAT CGA TG	

SDM_Ste2A265T_266F_5	GATAATATTCATCCTCACATTCAGTTTGAAACCAAACCAGGG	Change position 266 in *STE2* A265T to F
SDM_Ste2A265T_266F_3	CCCTGGTTTGGTTTCAAACTGAATGTGAGGATGAATATTATC	

Supplementary Table 4 : Codons used for saturation mutagenesis of site 290 in Ste2

**Amino acid**	**Codon**	**Amino acid**	**Codon**
**P**	CCA	**A**	GCT
**L**	CTA	**V**	GTT
**W**	TGG	**R**	AGA
**C**	TGT	**K**	AAA
**Y**	TAT	**N**	AAT
**S**	TCT	**T**	ACT
**F**	TTT	**M**	ATG
**G**	GGT	**I**	ATT
**E**	GAA	**Q**	CAA
**D**	GAT	**H**	CAT

SUPPLEMENTARY REFERENCES

1. Bashor CJ, Helman NC, Yan SD, Lim WA. Using engineered scaffold interactions to reshape MAP kinase pathway signaling dynamics. *Science* **319**, 1539-1543 (2008).

2. Di Roberto RB, Chang B, Trusina A, Peisajovich SG. Evolution of a G protein-coupled receptor response by mutations in regulatory network interactions. *Nature communications* **7**, 12344 (2016).
